# LygA retention on the surface of *Listeria monocytogenes* via its interaction with wall teichoic acid modulates bacterial homeostasis and virulence

**DOI:** 10.1371/journal.ppat.1011482

**Published:** 2023-06-28

**Authors:** Hao Yao, Guo Li, Xianglian Xiong, Fanxin Jin, Sirui Li, Xinyu Xie, Dan Zhong, Renling Zhang, Fanzeng Meng, Yuelan Yin, Xin’an Jiao

**Affiliations:** 1 Jiangsu Key Laboratory of Zoonosis, Yangzhou University, Yangzhou, China; 2 Key Laboratory of Prevention and Control of Biological Hazard Factors (Animal Origin) for Agrifood Safety and Quality, MOA of China, Yangzhou, Jiangsu Province, China; 3 Joint International Research Laboratory of Agriculture and Agri-product Safety of the Ministry of Education, Yangzhou, Jiangsu Province, China; 4 Jiangsu Co-Innovation Center for Prevention and Control of Important Animal Infectious Disease and Zoonoses, Yangzhou, Jiangsu Province, China; University of Tubingen, GERMANY

## Abstract

Wall teichoic acid (WTA) is the abundant cell wall-associated glycopolymer in Gram-positive bacteria, playing crucial roles in surface proteins retention, bacterial homeostasis, and virulence. The WTA glycosylation of *Listeria monocytogenes* is essential for surface anchoring of virulence factors, whereas the nature and function of the noncovalent interactions between cell wall-associated proteins and WTA are less unknown. In this study, we found that galactosylated WTA (Gal-WTA) of serovar (SV) 4h *L*. *monocytogenes* plays a key role in modulating the novel glycine-tryptophan (GW) domain-containing autolysin protein LygA through direct interactions. Gal-deficient WTA of Lm XYSN (Δ*galT*) showed a dramatic reduction of LygA on the cell surface. We demonstrated that LygA binds to Gal-WTA through the GW domains, and the binding affinity is associated with the number of GW motifs. Moreover, we confirmed the direct Gal-dependent binding of the GW protein Auto from the type I WTA strain, which has no interaction with rhamnosylated WTA, indicating that the complexity of both WTA and GW proteins affect the coordination patterns. Importantly, we revealed the crucial roles of LygA in facilitating bacterial homeostasis as well as crossing the intestinal and blood-brain barriers. Altogether, our findings suggest that both the glycosylation patterns of WTA and a fixed numbers of GW domains are closely associated with the retention of LygA on the cell surface, which promotes the pathogenesis of *L*. *monocytogenes* within the host.

## Author summary

Wall teichoic acid (WTA) of *Listeria monocytogenes* is involved in several key physiological functions through direct interaction with canonical virulence factors on the bacterial surface. However, how WTA modifies the retention of *Listeria* surface proteins and contributes to virulence remains unclear. Here, we found Gal-WTA mediates the anchoring of a novel autolysin LygA in SV 4h *L*. *monocytogenes* through interaction with the C-terminal GW domains. Both the WTA glycosylation and the composition of GW domains are necessary in the surface association of GW proteins. LygA facilitates bacteria to break through the intestinal barrier and blood-brain barrier. Collectively, our findings reveal that the specific interactions between WTA and GW-containing protein LygA contribute to *L*. *monocytogenes* homeostasis and pathogenesis.

## Introduction

*Listeria monocytogenes* is an important foodborne zoonotic pathogen largely relying on the proteins anchored to its cell surface for interacting with the environment, including infecting the host [1]. Surface proteins are mainly divided into three classes [1]: (i) covalently bound proteins, attach to the cell wall/peptidoglycan through the activity of a sortase enzyme, which recognizes an LPXTG sorting motif in the C-terminus. Some of these proteins are members of the internalin family. For instance, InlA triggers E-cadherin-mediated *L*. *monocytogenes* internalization in epithelial cells [2,3]; (ii) membrane-anchored proteins consist of hydrophobic tail proteins and lipoproteins. For instance, ActA relies on the hydrophobic domain of carboxyl terminus to link to the bacterial surface. Lipoprotein OppA attaching to the cell surface through a phospholipid-anchored N-terminal domain is involved in the intracellular survival of *L*. *monocytogenes* in macrophages [4]; (iii) surface proteins, including glycine-tryptophan (GW) proteins and LysM proteins, link to the cell wall through noncovalent interactions mediated by C-terminal domains. For instance, Ami, Auto, and IspC have GW modules at the C-terminus [5–7], while P60 and MurA carry LysM domains [8]. All these proteins are autolysins and dependent on the C-terminal anchoring domains for attaching to the bacterial surface. The surface proteins in Gram-positive bacteria exert their functions by anchoring to the peptidoglycan or teichoic acids (TAs).

Wall teichoic acid (WTA) plays key roles in the regulation of cell morphology and division, autolysis, resistance to antimicrobial peptides, and virulence [9–11]. WTA consists of two parts: a disaccharide-linked unit and a main chain polymer consisting of a phosphodiester-linked polyol repeats [9]. The diversity of structures is mainly attributed to the presence of glycosyl substituents, d-alanyl esters, and/or repeating units [12,13]. Two structural classes of WTA exist in *L*. *monocytogenes* that are mainly distinguished by their variable repeating units into type I (serovars [SVs] 1/2, 3, and 7) or type II (SVs 4, 5, and 6) WTA [14]. Notably, the WTA of SV 4b strains, associated with most outbreaks of listeriosis, is decorated with galactose (Gal) and glucose (Glc) [14,15]. Recent studies have demonstrated that the l-rhamnosylation or galactosylation of WTA contributes to virulence by promoting efficient surface association of the virulence proteins InlB and Ami through GW domains [16–18]. The GW motif is present in various Gram-positive bacteria and is responsible for anchoring proteins to the cell wall [19]. Several surface autolysins, such as *L*. *monocytogenes* Ami, *Staphylococcus epidermidis* AtlE, *S*. *saprophyticus* Aas, and *Streptococcus suis* AtlA_SS_, have been reported to contribute to bacterial virulence through their GW repeat modules [20–22]. In *L*. *monocytogenes*, approximately 5% of proteins are thought to be located on the surface [23]. However, the anchoring mechanism of proteins to WTA on the cell surface remains poorly understood.

The newly defined SV 4h *L*. *monocytogenes* XYSN with unique galactosylated WTA, GlcNAc residues decorated with galactose bounding at the C-4 position of the ribitolphosphate polymer chain, had caused outbreaks of ovine listeriosis [16]. We previously demonstrated that Gal-WTA promotes the adherence of the virulence proteins Ami and ActA to the cell wall and plays a crucial role in helping *L*. *monocytogenes* break through the intestinal barrier and successfully colonize organs [16]. In this study, we found that Gal-WTA is also a prerequisite for the stable retention of other surface proteins, including GW-containing proteins. Based on liquid chromatography-tandem mass spectrometry (LC-MS/MS) analysis of surface proteins, we identified a novel autolysin with a lysozyme subfamily 2 (LYZ2) domain and six GW domains (LygA), which was predominantly released (24-fold) from the cell surface in the galactosylation-deficient mutants. We also demonstrated the complexity of the GW domains and the glycosylation modification of WTA are necessary to mediate the GW proteins surface binding. Importantly, our findings unraveled that autolysin LygA, binding to Gal-WTA through the fixed number of GW domains, facilitates bacterial autolysis and virulence.

## Results

### The surface association of LygA protein is modulated by galactosylated WTA

**Fig 1:**
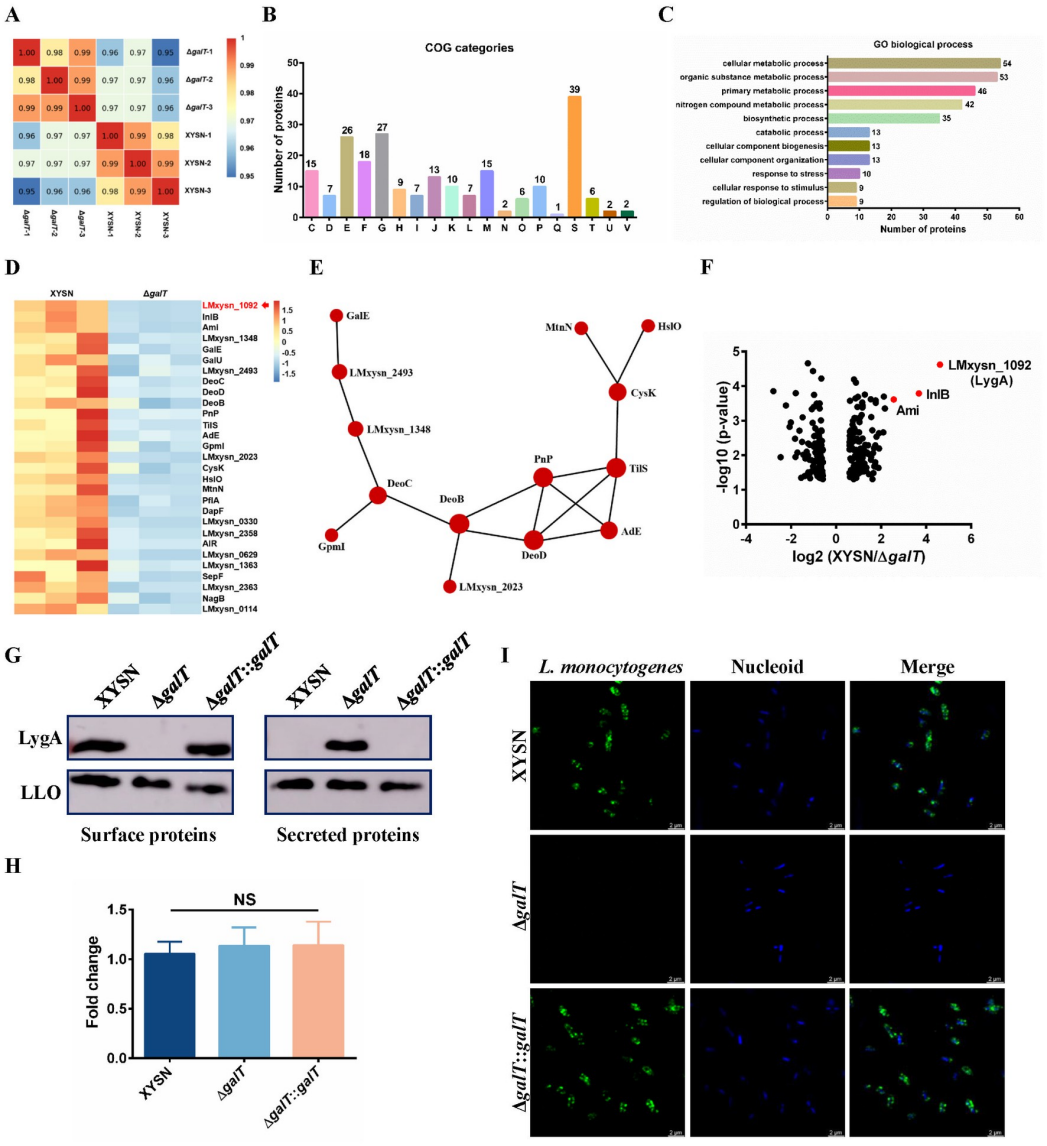
Analysis of changes in the cell surface proteome between *L*. *monocytogenes* XYSN and Δ*galT* mutant. (A) Heat map of Pearson’s correlation coefficient for protein quantification between any two samples. Pearson’s coefficient closer to -1 represents a negative correlation, closer to 1 represented a positive correlation, and closer to 0 represented uncorrelation. (B) COG functional classification distribution map obtained from the surface proteome of WT and Δ*galT* mutant. The COG abbreviations were as follows: [C] Energy production and conversion; [D] Cell cycle control, cell division and chromosome partitioning; [E] Amino acid transport and metabolism; [F] Nucleotide transport and metabolism; [G] Carbohydrate transport and metabolism; [H] Coenzyme transport and metabolism; [I] Lipid transport and metabolism; [J] Translation, ribosomal structure and biogenesis; [K] Transcription; [L] Replication, recombination and repair; [M] Cell wall/membrane/envelope biogenesis; [N] Cell motility; [O] Posttranslational modification, protein turnover and chaperones; [P] Inorganic ion transport and metabolism; [Q] Secondary metabolite biosynthesis, transport and catabolism; [S] Function unknown; [T] Signal transduction mechanisms; [U] Intracellular trafficking, secretion and vesicular transport; [V] Defense mechanisms. (C) Top 11 prevalent biological processes from differential abundant proteins. (D) Heat map showed relatively significant changes in abundance of the 29 proteins. Red indicated a relative increase in the amount of protein, and blue represented a relative decrease. (E) Analysis of proteins interaction. Red represented up-regulated expression of proteins. The size of the circle represents the number of differential proteins and their interacting proteins. (F) Volcano plot revealed the fold change (> 1.5) and statistical significance (*P* < 0.05) in abundance of all identified proteins in the WT and Gal-deficient strains. (G) Western blot analysis of surface and secreted *L*. *monocytogenes* proteins obtained from XYSN, Δ*galT*, and Δ*galT*::*galT* strains. LLO protein levels were used as a loading control. Proteins were detected using LygA polyclonal antibody and monoclonal antibody against LLO (3B6). Protein levels were normalized using the BCA Protein Assay Kit (Beyotime). The image presented was representative of three individual experiments. (H) Quantitative real-time PCR analysis of *lygA* expression in the XYSN, Δ*galT*, and Δ*galT*::*galT* strains. Error bars represent SD; n = 3 independent experiments. Statistical analyses were performed by Tukey’s multiple comparisons test; ns: no significance. (I) Confocal images of XYSN, Δ*galT*, and Δ*galT*::*galT* strains, which were subsequently stained with anti-LygA polyclonal antibody (primary antibody) and Alexa Fluor 488-conjugated anti-mouse antibody (green). The nucleoid was stained using DAPI (blue). Magnification of all images: 6000×. Scale bars, 2 μm.

To identify novel surface proteins potentially associated with the cell wall through WTA galactosylation, label-free quantitative proteomic analysis of surface proteins from the *L*. *monocytogenes* XYSN and Δ*galT* strains (a LMxysn_*1095* gene mutant of *L*. *monocytogenes* XYSN with Gal-deficient WTA) was performed [16]. A total of 1579 proteins were identified (S1 Table). Of these, 229 proteins had significantly different expression levels (fold-change > 1.5), 134 proteins were up-regulated and 95 proteins were down-regulated. The total proteome showed high reproducibility in three biological replicates (Fig 1A), indicating relatively reliable results of proteomic analysis.

All proteins whose levels were significantly altered were assigned to 19 processes according to the results of Clusters of Orthologous Groups of proteins (COG) analysis (Fig 1B). LMxysn_*1095* encodes a galactosyltransferase (GalT) that is responsible for WTA galactosylation [16]. Therefore, the enriched differential proteins were relatively more involved in carbohydrate/amino acid/nucleotide transport and metabolism as well as in cell wall/membrane/envelope biogenesis. The effects of metabolic process and stress response were also revealed in the results of the 11 most highly enriched Gene Ontology (GO) biological processes (Fig 1C). 29 down-regulated proteins with significant changes were selected in Δ*galT* mutant to generate a heat map. Of which, GalE, GalU, LMxysn_1348, and LMxysn_2493 were related to the galactose metabolism; DeoD, PnP, and AdE were associated with nicotinate and nicotinamide metabolism (Fig 1D). Moreover, the protein-protein interaction (PPI) network was constructed according to the STRING (v.10.5) protein network interaction database, further demonstrating that these proteins may participate in the synthesis of WTA galactosylation (Fig 1E). The surface virulence proteins InlB and Ami were also enriched, with 12.76-fold and 5.89-fold changes being noted in their abundance, respectively. The change of ActA protein was not enriched. We further verified the surface distribution of ActA by Western blot. Consistently, the retention of ActA on the cell wall surface was associated with galactosylated WTA (S1 Fig). Remarkably, a previously uncharacterized LMxysn_1092 protein, LygA, was identified to have the most significant change in abundance according to the proteome database of SV 4b *L*. *monocytogenes* F2365 (24.42-fold) (Fig 1F, S2 Table).

Considering the decrease in abundance of LygA on the cell surface of the galactosylation-deficient strain, we extracted surface and secreted proteins from exponential cultures of the XYSN, Δ*galT*, and Δ*galT*::*galT* strains and further verified the results by performing Western blot analysis using LygA polyclonal antibody. Consistently, we observed the redistribution of LygA from the cell surface to the secreted compartment (Fig 1G). Moreover, the transcript levels of *lygA* did not change, as revealed by the results of qRT-PCR (Fig 1H). To further verify the co-localization, confocal microscopy using LygA polyclonal antibody was carried out. The results revealed that LygA was distributed exclusively and was unevenly localized in clusters on the cell surface of wild-type (WT) and complemented strains, but not in the Δ*galT* mutant (Fig 1I). Taken together, these results indicate that WTA galactosylation is crucial for the efficient association of LygA to the bacterial surface.

### GW domains of LygA interact with WTA in a Gal-dependent manner

**Fig 2:**
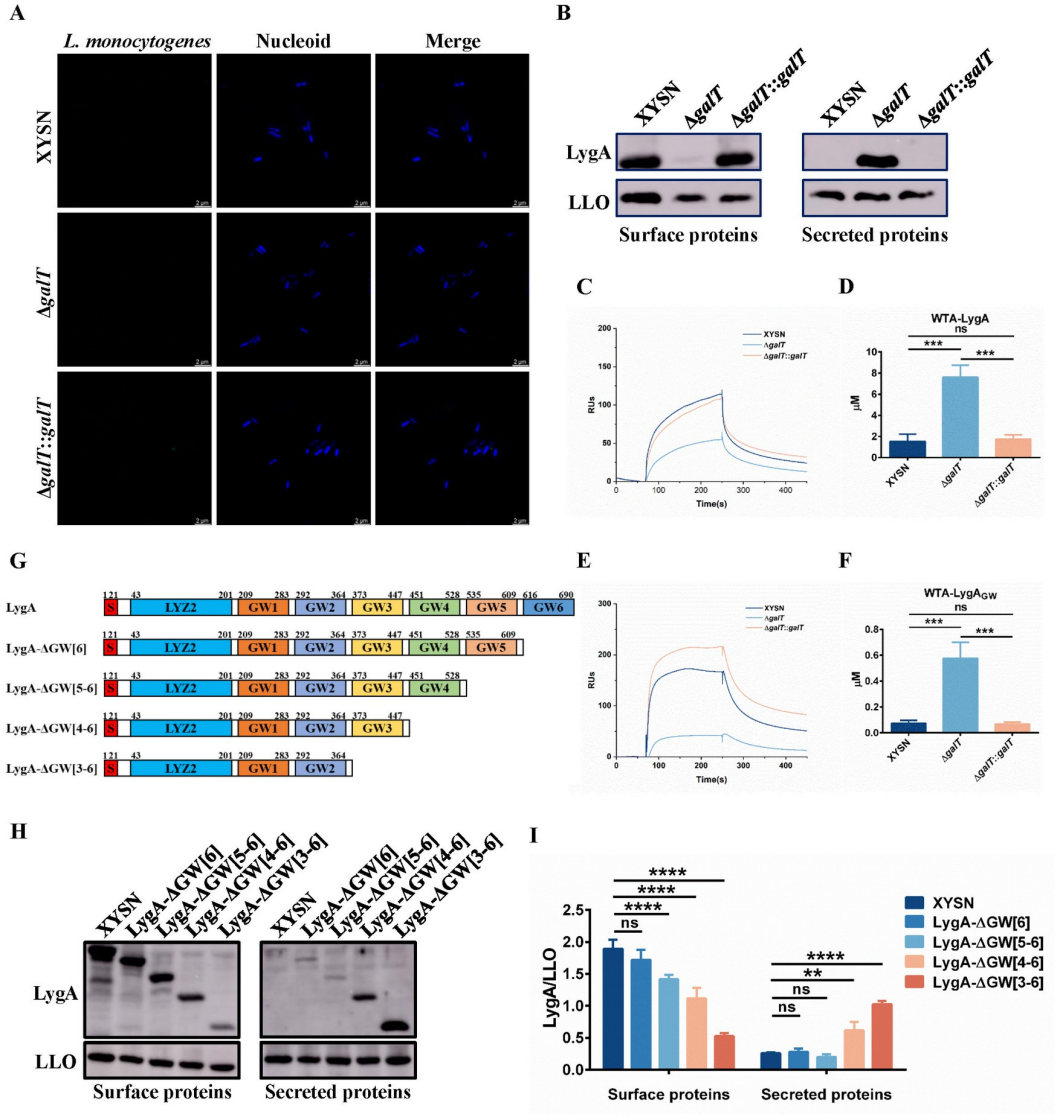
LygA interacts with Gal-WTA through its GW domain. (A) Confocal images of bacteria. XYSN, Δ*galT*, and Δ*galT*::*galT* cells were subsequently labeled with anti-LygA_GW_ polyclonal antibody and Alexa Fluor 488-conjugated anti-mouse antibody (green), and DNA was stained with DAPI (blue) following fixation of the sample. Magnification of all images: 6000×. Scale bars, 2 μm. (B) The surface and secreted proteins of *L*. *monocytogenes* obtained from XYSN, Δ*galT*, and Δ*galT*::*galT* strains were analyzed by Western blot using LLO protein levels as a control. Proteins were detected with LygA_GW_ antibody or monoclonal antibody against LLO (3B6). (C, E) Association of LygA or LygA_GW_ proteins with purified WTA polymers isolated from XYSN, Δ*galT*, and Δ*galT*::*galT* strains by SPR analysis. The concentrations of LygA/LygA_GW_ and WTA/Gal-WTA were 0.2 mg/mL and 3.13 μM, respectively. RUs: relative units. (D, F) Assessment of interactions between LygA or LygA_GW_ proteins and WTA at concentrations ranging from 0 to 25 μM to determine the binding affinity response. K_D_ was determined through three independent experiments; error bars represent SD. Statistical analyses were performed by Tukey’s multiple comparisons test. ***P < 0.001; ns: no significance. (G) Schematic of the deletion fragments of LygA in this study. (H, I) Western blot analysis of the effect of GW domain numbers on LygA anchoring to the cell wall surface and the relative content of LygA to LLO in surface proteins and secreted proteins. Error bars represent SD; n = 3 independent experiments. Statistical analyses were performed by Dunnett’s multiple comparisons test; **P < 0.01; ****P < 0.0001; ns: no significance.

InlB and Ami have been demonstrated to associate with cell wall components by noncovalent bonding through the C-terminal domain containing GW repeats [18]. Western blot analysis indicated that Gal-WTA promoted the association of LygA with the *L*. *monocytogenes* cell surface. Therefore, we further observed the role of GW domains in LygA anchoring through the confocal microscopy. As expected, the localization of LygA was also not observed on the cell surface of WT strains using LygA_GW_ polyclonal antibody, which can be used to label LygA in Western blot analysis (Fig 2A and 2B). These results suggest that GW repeats are buried in the cell wall and Gal-WTA is essential for the accurate localization of LygA.

Sumrall *et al*. demonstrated that TAs are associated directly with InlB through the surface plasmon resonance (SPR) analysis [17]. Therefore, whether the interaction between LygA and WTA also occurs in a Gal-dependent manner was evaluated. We successfully obtained different types of WTA and produced recombinant LygA and LygA_GW_ proteins (S2A and S2B Fig). Notably, the SPR assay revealed that Gal-WTA strongly interacted with LygA; however, the binding ability was strikingly attenuated in galactosylation-deficient WTA (Figs 2C and S2C). Consistently, the binding affinity of Gal-WTA to LygA (K_D_ = 1.54 μM) was stronger than that of galactosylation-deficient WTA (K_D_ = 7.61 μM) (Fig 2D). As expected, the interaction of LygA_GW_ with Gal-WTA showed the same trend as that of LygA (Figs 2E and 2F and S2D). These results suggest that the retention of LygA on the cell surface is mediated by WTA through the GW domains in a Gal-dependent manner.

GW domain proteins are non-covalently anchored to the cell wall surface via the fixed numbers of GW motifs, such as InlB (3 GW domains), Auto (4 GW domains), and Ami (8 GW domains), whereas the role of the number of GW motifs remains largely unclear. Thus, we sequentially knocked out different numbers of GW domains at the carboxyl terminus of LygA protein (Fig 2G), and the content of the different LygA truncates in surface and secreted proteins was analyzed using Western blot. The results showed that the anchoring of LygA on the cell wall surface was hardly affected when one GW domain was absent. However, LygA was gradually shed from the cell wall surface as the number of GW domains continued to decrease, especially LygA almost lost its anchoring ability when the four GW domains were deleted (Fig 2H and 2I). These data indicate that the non-covalent binding of LygA to the bacterial surface is coordinately modulated by a combination of fixed GW domains.

It is known that l-rhamnosylation of WTA does not affect the association of Auto with the cell surface [18]. Therefore, we determined whether the galactosylation of WTA played the same role in the association of Auto with GW domains. We produced recombinant Auto from EGD-e (S3A Fig) and assessed whether it could directly interact with Gal-WTA by SPR analysis. Unexpectedly, the affinity results revealed that Gal-WTA polymers associated strongly with Auto, while galactosylation-deficient WTA polymers showed a dramatically lower binding ability (S3B and S3C Fig). Similarly, the K_D_ value of binding affinity was significantly higher in galactosylation-deficient WTA than in the WT (S3D Fig). In addition, l-rhamnosylated WTA couldn’t bind with LygA and Auto (S3E and S3F Fig). All together, these data indicate that the glycosylation patterns of WTA are important for the surface association of GW proteins.

### LygA has peptidoglycan hydrolase activity

**Fig 3:**
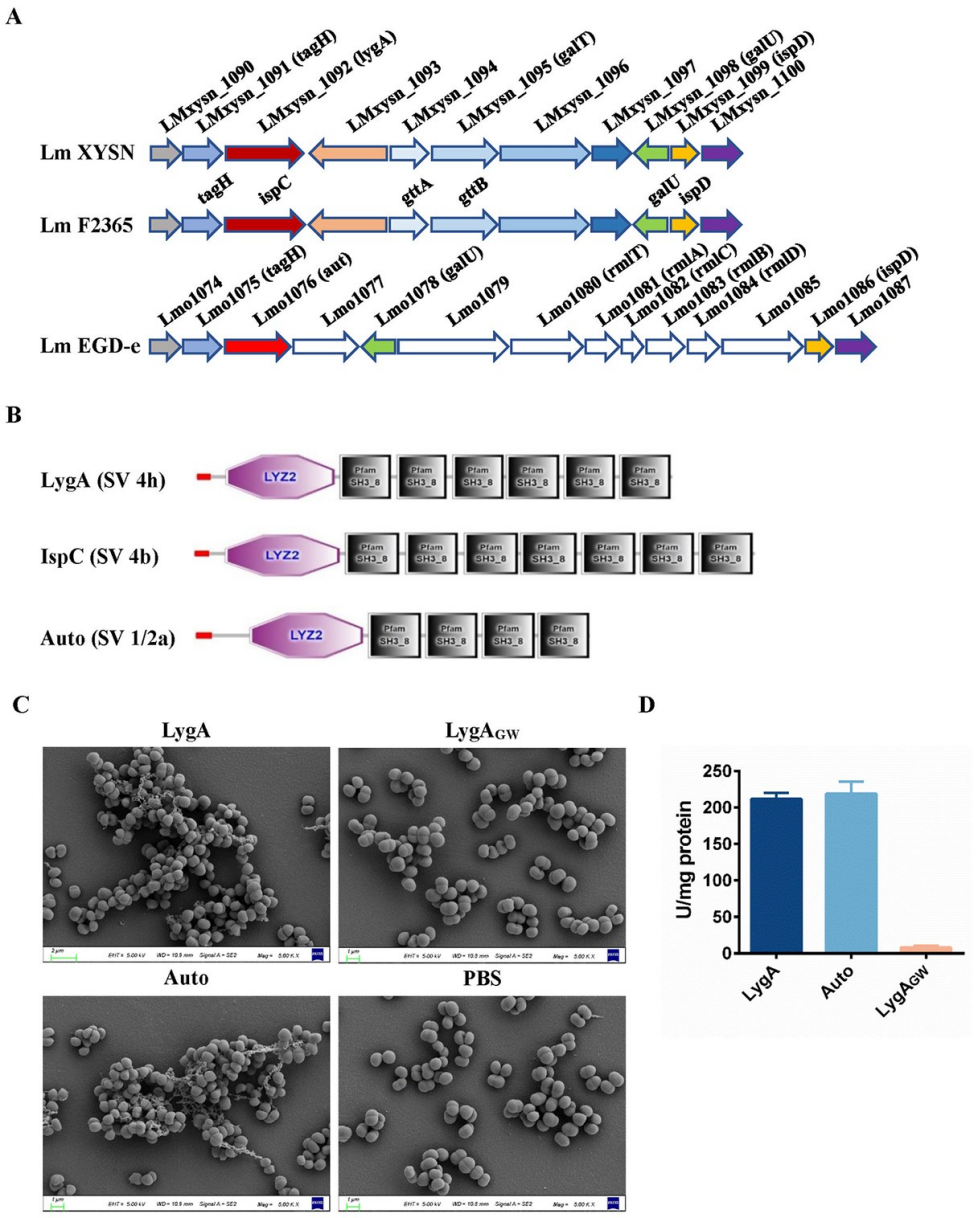
Genomic analysis and functional prediction. (A) Genetic organization of gene clusters in SV 4h XYSN, SV 4b F2365, and SV 1/2a EGD-e strains. Arrows indicated genes and transcription directions. Genes with the same color represented their products, i.e., the same proteins. (B) Analysis of LygA, IspC, and Auto autolysin domains with SMART. All of them possessed a signal peptide, an LYZ2 domain, and four to seven GW motifs. (C) Observation of bacterial morphology through scanning electron microscope. The *M*. *lysodeikticus* cells on glass slides were incubated with 10 μg/mL of LygA, Auto, or LygA_GW_ proteins. (D) Detection of the lytic activity of LygA by turbidity spectrophotometry. The different recombinant proteins were added to the sensitized *M*. *lysodeikticus* cell suspension and incubated for 30 min. The difference in the OD_600_ value between this suspension and the corresponding control was used to calculate the lytic activity. Error bars represent SD; n = 3 independent experiments.

LygA consisting of 693 amino acids is 92.45% identical to IspC in SV 4b strains and 39.60% identical to Auto in SV 1/2a strains, according to genomic and bioinformatic comparisons. It is worth noting that the gene *lygA* of Lm XYSN, *ispC* of Lm F2365, and *aut* of Lm EGD-e are adjacent to the corresponding gene cluster responsible for WTA glycosylation (Fig 3A) [10,16,17]. Moreover, analysis of protein domain architectures using the Simple Modular Architecture Research Tool (SMART) revealed that all three proteins possess the LYZ2 and GW domains (Fig 3B). The LYZ2 domain has been shown to hydrolyze peptidoglycan [24]. These results imply that LygA might have similar functions to IspC, which is an autolysin protein with peptidoglycan hydrolase activity.

Subsequently, we studied the hydrolase activity of LygA using *M*. *lysodeikticus* cells by turbidity spectrophotometry and observed the bacterial morphology with scanning electron microscope. After the sensitized cells on glass slides were incubated with LygA, the cells gathered into larger clusters compared with the LygA_GW_ treatment group, which may be due to the function of LygA to change the cell surface hydrophobicity. Autolysin Auto showed similar results to LygA (Fig 3C). Moreover, LygA possessed a strong synergistic effect as Auto with an activity of 211 U, while LygA_GW_ had no hydrolase activity (Fig 3D). These results strongly suggest that LygA is a novel autolysin protein in *L*. *monocytogenes*.

### LygA plays crucial roles in bacterial autolysis and cell division

**Fig 4:**
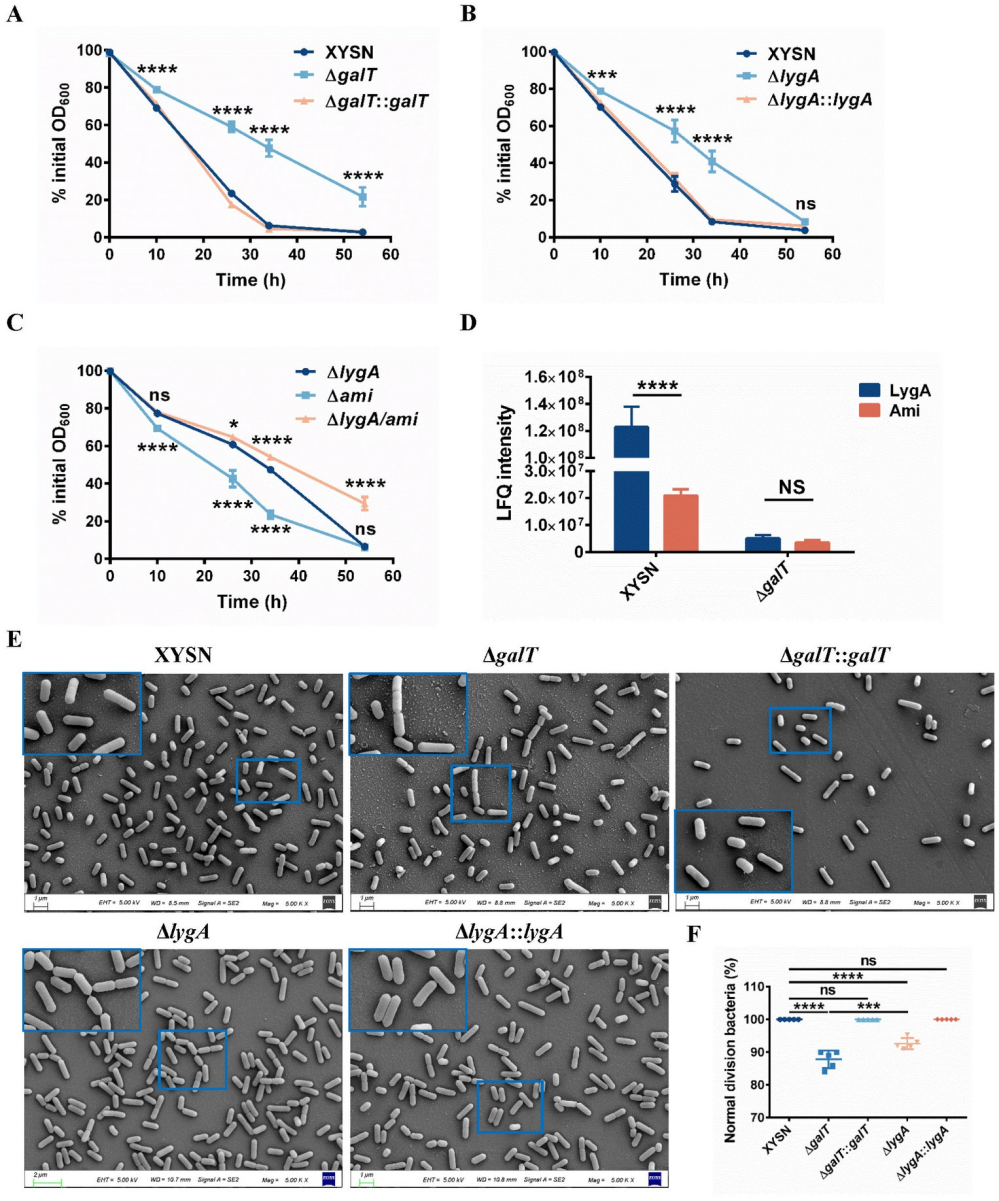
LygA affects autolytic activity and cell division. (A, B, and C) XYSN, Δ*galT*, Δ*galT*::*galT*, Δ*lygA*, Δ*lygA*::*lygA*, Δ*ami*, and Δ*lygA*/*ami* cells were washed and resuspended in glycine buffer (50 mM, pH 8.0). The OD_600_ values were adjusted to 1.00 and monitored at each time point until clarification. Error bars denote SD; n = 3 independent experiments. Statistical analyses were performed by Tukey’s multiple comparisons test. *P < 0.05; ***P < 0.001; ****P < 0.0001; ns: no significance. (D) Abundance of LygA and Ami proteins on the cell surface was determined by proteomics analysis. Label-free quantification (LFQ) intensity of protein was used as protein quantitative data to compare the difference of protein expression between the experimental group and the control group. Error bars denote SD. Statistical analyses were performed by Sidak’s multiple comparisons test. ****P < 0.0001; ns: no significance. (E) Scanning electron microscopy images depicting the division of listerial cells. (F) The number of abnormally dividing daughter bacteria was counted as the percentage of the total number of cells. Each dot represents one SEM field of bacteria. Statistical analyses were performed by Tukey’s multiple comparisons test. ***P < 0.001; ****P < 0.0001; ns: no significance.

Cell wall glycopolymers (CWGs), such as WTA, have highly variable structures and are densely functionalized in various processes occurring in the cell envelope of Gram-positive bacteria. LygA with peptidoglycan hydrolase activity may affect the autolysis of bacteria. Therefore, we assessed the autolysin activity of WT XYSN and an isogenic mutant strain devoid of Gal-WTA. Suspensions of the XYSN and complemented strains became clear at 54 h, while the Δ*galT* mutant suspension still showed a turbid state. The Δ*galT* strain showed significantly lesser lysis than the WT strain, as per the OD_600_ values. The phenotype was reverted in the Δ*galT*::*galT* strain (Fig 4A). Moreover, the autolytic activity of the *lygA*-deleted strain significantly decreased within 50 h (Fig 4B). Ami, another autolysin associated with cell surface mediated by glycosylation of WTA, is also related to bacterial autolysis activity [18]. Notably, the physiological levels of autolysis in Δ*lygA* strain were dramatically decreased compared with Δ*ami* strain, while the Δ*ami*/*lygA* mutant was more attenuated regarding autolysin activity than Δ*lygA* strain (Fig 4C). On the other hand, LygA was far more abundant than Ami on the cell surface, as revealed by quantitative proteomic analysis (Fig 4D). Altogether, these data indicate that autolysin LygA and Ami are related to the autolysis activity of *L*. *monocytogenes*. In particular, LygA dominates the physiological process of autolysis.

Autolysins, such as P60 and MurA, are responsible for cell separation [25,26]. Both MurA and IspC are *N-*acetylglucosaminidases that function as cell wall hydrolases [27]. Therefore, using scanning electron microscopy, we also observed the cell division ability of the bacteria. Interestingly, 12.2% bacterial cells of Δ*galT* mutant were unable to effectively separate during cell division and form longer chains. Moreover, the same phenomenon was observed in Δ*lygA* mutant (7.4%), while the WT strain and the revertant strain divided normally (Fig 4E). In addition, more cells were failing to divide normally in the Δ*galT* mutant than in the Δ*lygA* strain (Fig 4F). The phenomena imply that LygA, an autolysin anchored to the bacterial surface through the Gal-WTA, promotes cell division in *L*. *monocytogenes*.

### LygA is required for the invasion and virulence of *L*. *monocytogenes*

**Fig 5:**
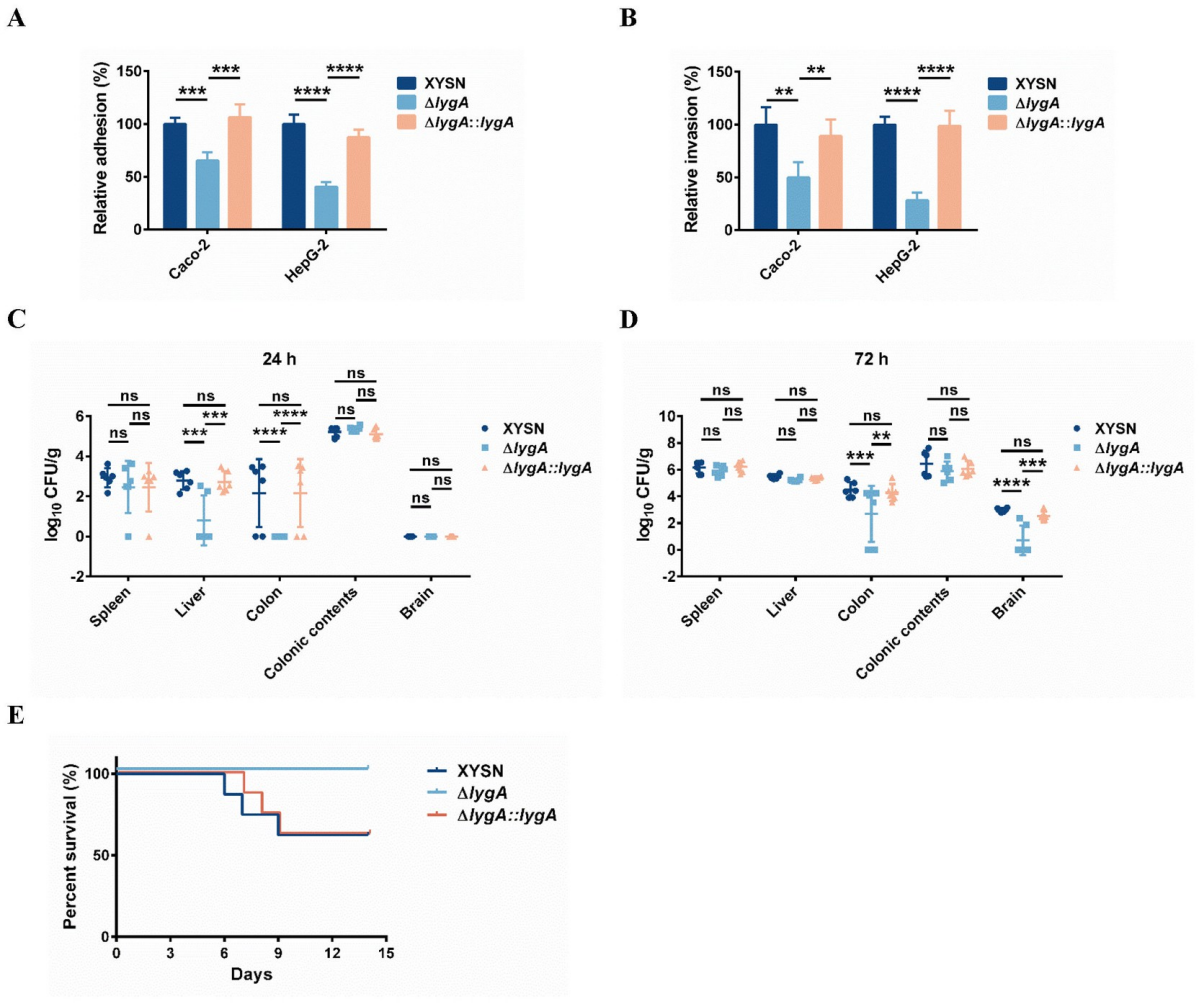
LygA contributes to bacterial adhesion and infection *in vitro* and *in vivo*. Quantitative analysis of adhesion (A) and invasion (B) of the Δ*lygA* mutant in various eukaryotic cell lines in comparison with the WT strain. Cells were infected with XYSN, Δ*lygA*, and Δ*lygA*::*lygA* strains at an MOI of 20 for 1 h and incubated for another 15 min. The number of bacteria released from the cells was quantified by plating the lysates. The quantity of Δ*lygA* was calculated relative to that of the WT (100 %). Error bars represent SD; n = 3 independent experiments. Statistical analyses were performed by Tukey’s multiple comparisons test. **P < 0.01; ***P < 0.001; ****P < 0.0001; ns: no significance. (C) and (D) Comparison of organ colonization properties among the XYSN, Δ*lygA* and Δ*lygA*::*lygA* strains. The infection dose was 3×10^6^ CFU. Experiments were performed at 24 and 72 h post-infection. Each dot represents an organ from an infected mouse. Log CFUs/g represents the mean of six mice per group. Error bars represent SD. Data were obtained from two independent experiments. Statistical analyses were performed by Tukey’s multiple comparisons test. **P < 0.01; ***P < 0.001; ****P < 0.0001; ns: no significance. (E) Survival curves of female C57BL/6 mice (n = 8/group) were determined via orogastric inoculation of 3 × 10^6^ CFU of XYSN, Δ*lygA*, and Δ*lygA*::*lygA*.

Both Auto and IspC contribute to bacterial entry into eukaryotic cells [6,28]. Therefore, we assayed whether LygA was involved in the invasion capability of *L*. *monocytogenes*. To determine the role of LygA in infecting eukaryotic cells, we assessed the adhesion and invasion abilities of the XYSN, Δ*lygA*, andΔ*lygA*::*lygA* strains using Caco-2 and HepG-2 cells. The mutant strain exhibited impaired adhesion and invasion abilities in comparison with the WT strain. Moreover, the adhesive and invasive phenotypes were restored in the complemented strain (Fig 5A and 5B), indicating that LygA plays an important role in *L*. *monocytogenes* infection of intestinal epithelial cells and hepatocytes.

Finally, we evaluated the contribution of LygA to the virulence potential of *L*. *monocytogenes* XYSN *in vivo* using the mouse intragastric infection model. The deficiency of LygA in bacteria significantly reduced their invasion and colonization abilities in the liver and colon at 24 h post-infection (Figs 5C and S4A). Importantly, at 72 h post-infection, the multiplication of the mutant was further impaired in the colon and brain; however, the counts of all the three strains were similar in the spleen and liver, as well as in colonic contents (Figs 5D and S4B). In addition, there were no differences in the growth curves of each strain (S4C Fig). We further examined the virulence potential of LygA through the survival curves. C57BL/6 mice were orogastrically inoculated with XYSN, Δ*lygA* and Δ*lygA*::*lygA* at a dose of 3×10^6^ CFU. Three infected mice succumbed to infection with XYSN and Δ*lygA*::*lygA* between days 6 and 9, respectively. No deaths were recorded for Δ*lygA* mutant at the same dose (Fig 5E). Altogether, these results confirm that LygA is required for bacterial virulence and plays crucial roles in the early colonization of the intestine and further infection of the host.

## Discussion

**Fig 6:**
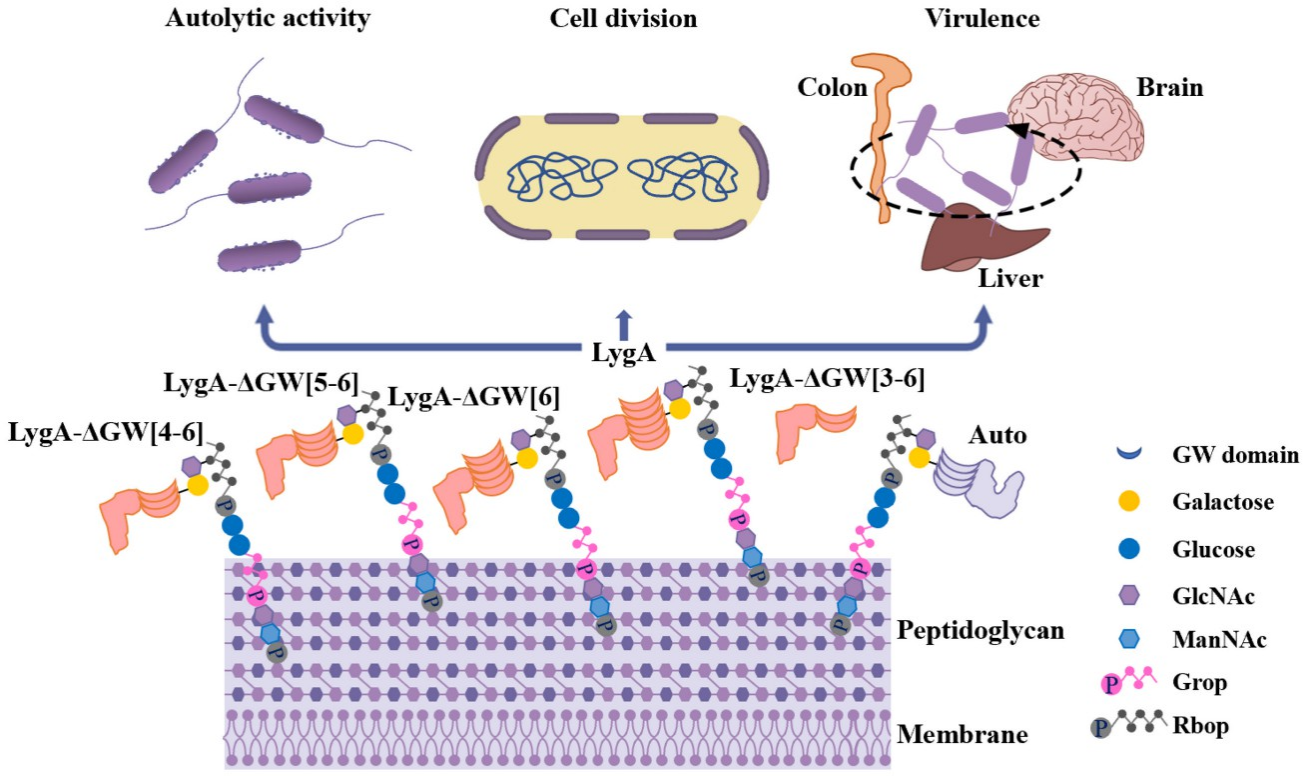
Function and surface anchoring mechanism of LygA. The LygA protein relies on galactose-modified WTA and GW domains binding to the cell surface. As the number of GW domains decreases, the binding ability of LygA to the cell wall surface is weakened which in turn leads to the shedding of LygA. In addition, Auto in SV 1/2 strains also directly interacts with Gal-WTA. As an autolysin protein, LygA is involved in bacterial autolytic activity, cell division, and then mediates the virulence of *L*. *monocytogenes*.

The cell wall of Gram-positive bacteria acts as a surface organelle that provides anchor sites for numerous surface proteins, thereby interacting with the environment and contributing to the invasion of host cells [29,30]. The WTA of *L*. *monocytogenes* is a cell wall component that is required for the stable localization of important virulence proteins on the bacterial surface. In this study, we employed quantitative proteomics to explore the *L*. *monocytogenes* surface proteins associated with WTA galactosylation. We revealed a novel autolysin, LygA, that binds to WTA in a Gal-dependent manner through GW motifs, and is involved in remodeling of the bacterial cell wall and virulence (Fig 6).

GW proteins play crucial roles in bacterial growth, division, and infection. Currently, among the reported GW-containing proteins of *L*. *monocytogenes* [1,5], only InlB, Ami, and Auto are strongly associated with the cell surface [18]. Previous studies have confirmed that l-rhamnosylated WTA of the EGD-e strain could promote the surface association of InlB and Ami through GW domains. Consistently, Gal-WTA helps SV 4 strains retain InlB and Ami through GW domains [16–18]. In this study, we found that impaired galactosylation of WTA led to the release of multiple *L*. *monocytogenes* GW proteins, such as InlB and Ami, from the surface of SV 4h strains. Notably, GW-containing LygA, a novel autolysin protein with peptidoglycan hydrolase activity, was most dramatically released (24-fold) from the cell surface to the culture medium in galactosylation-deficient mutant. We confirmed that the interaction of Gal-WTA with GW domains promotes the efficient surface anchoring of LygA and emphasized the fixed numbers of GW motifs coordinate to regulate the binding of GW proteins to the cell wall surface. Considering these findings, we confidently propose that the retention of LygA on the bacterial surface is dependent on the WTA galactosylation. TAs facilitate the association of GW proteins with the cell surface in various Gram-positive bacteria. LytA and PspA of *S*. *pneumoniae* are anchored to the choline residues of lipoteichoic acid depending on the C-terminal choline-binding repeat domains containing 20 amino acids [31,32]. However, the GW domain of *L*. *monocytogenes* contains 80 amino acids, suggesting that the coordination patterns are related to the complexity of both WTA and GW protein structures.

*L*. *monocytogenes* contains two types of WTA: (i) type I WTA consists of repeating units of ribitol phosphate, (ii) type II WTA consists of β-*N*-acetylglucosamine (GlcNAc) residues incorporated as part of the ribitol phosphate polymer chain [14]. Moreover, the structural variation depends on the carbon position where GlcNAc is linked to ribitol phosphate as well as on further glycosylation or *O*-acetylation modifications. SV 1/2 strains belonging to type I WTA are decorated with α-GlcNAc and rhamnose. Conversely, in SV 4 strains, type II WTA is substituted with β-GlcNAc, glucose, and/or galactose [14]. Different glycosylation modifications on the main chain of WTA play various roles in the activities of bacteria. l-rhamnosylated WTA of the SV 1/2a EGD-e strain was found to have no association with the anchoring of Auto carrying GW domains [18]. Interestingly, we not only revealed that Gal-WTA interacts with LygA through GW domains but also unequivocally demonstrated the direct Gal-dependent binding of Auto to WTA. Therefore, our data strongly proved that Gal-WTA but not l-rhamnosylated WTA from the type I WTA strain could confer affinity to Auto proteins [18]. Notably, we found that the relative units (Rus) of Auto binding to Gal-WTA were strikingly greater than that of LygA binding to Gal-WTA at the same concentration of galactose-modified WTA, indicating that the affinity of Auto to Gal-WTA is higher than that of LygA to Gal-WTA. Auto contains two less GW motifs than LygA, and the homology of amino acids in GW domains is very low between Auto and LygA (S5 Fig). This result indicates that the structure of GW domains endowed by specific amino acids also affects the affinity of WTA and GW proteins. Consistent with a previous report that the retention of GW proteins on the cell wall could depend on the diverse glycosylation patterns and the composition of GW motifs [18]. Altogether, these observations suggest that the galactosylation of WTA plays a crucial role in the anchoring of cell surface proteins.

The structural and constitutional variations in WTA affect the autolytic activity of bacteria through interactions with autolysin proteins. In *S*. *aureus*, the d-alanylated WTA-deficient mutant Δ*dltA* significantly reduced autolytic activity due to the loss of endogenous autolysin [33]. The TA polymers of *Bacillus subtilis* were found to interact with LytE, an autolysin with hydrolytic peptidoglycan activity, thereby enhancing bacterial adaptability to different environments [34]. Moreover, a previous study confirmed that l-rhamnosylation-deficient strain showed slightly reduced autolytic activity because of the release of only the autolysin Ami [18]. Interestingly, we found that Gal-WTA deficient *L*. *monocytogenes* showed the release of both Ami and LygA. The autolysis activity of Δ*lygA* was significantly lower than that of Δ*ami*, suggesting that LygA plays a more important role than Ami during cell lysis and cell wall expansion. Previous research had revealed that WTA protects *S*. *aureus* from autolysis by targeting Atl to the septum [35]. We found that LygA was distributed in multiple regions of the cell wall, consistent with the patterns noted in bacilli through multipoint insertion and incorporation of new PGs along the lateral wall in a dispersed manner [36]. The cell division was also defective when LygA was released from the cell wall. Moreover, galactosylated WTA appeared to promote bacterial surface association of SepF according to the proteomic analysis (Fig 1D). SepF is essential for cell division, which potentially results in more cells cannot divide normally in the Δ*galT* mutant [37]. Hence, our findings demonstrate that the surface retention of LygA modulated by Gal-WTA is important for efficient bacterial autolysis and cell division.

Autolysins play crucial roles in Gram-positive bacterial infections and pathogenesis, for example, AtlE of *S*. *epidermidis* [38], LytA and LytB of *S*. *pneumoniae* [39,40], P60, Ami, Auto, and IspC of *L*. *monocytogenes* [6,28,41,42]. The LYZ2 domain-containing proteins Auto and IspC are peptidoglycan hydrolases, playing an essential role in the virulence of *L*. *monocytogenes*. We found that the LYZ2 domain-containing protein LygA promotes the adhesion, invasion, and colonization of *L*. *monocytogenes* in the intestine of mice via intragastric infection. Meningitis caused by Lm can largely lead to death in mice [43]. The colonization of Δ*lygA* mutant in mouse brain was significantly impaired. In addition, the survival curve results showed that three mice succumbed to infection with XYSN and Δ*lygA*::*lygA*, whereas no deaths were recorded for Δ*lygA* mutant using the same dose. Taken together, these results suggest that LygA plays a critical role in the virulence of *L*. *monocytogenes*. Notably, mice are not the natural susceptible hosts of *L*. *monocytogenes*. However, our previous study revealed that C57BL/6 mice are highly naturally susceptible to the emerging SV 4h isolates, with 200-400-fold higher virulence being noted than that of *L*. *monocytogenes* EGD-e via intragastric infection [16]. We further demonstrated that the galactose-based decoration of WTA in SV 4h strains and subsequent interaction with ActA and Ami could help *L*. *monocytogenes* cross the intestinal barrier [16]. These data suggest that the unique galactose-based decoration of WTA increases bacterial virulence by the stable localization of LygA as well as ActA and Ami, therefore enhancing SV 4h strains to break through the intestinal barrier and blood-brain barrier, successfully colonizing the intestine and brain of the host.

In conclusion, this study emphasizes the retention of surface protein LygA to the cell wall is entirely dependent on a fixed number of GW modules interacting with Gal-WTA. Importantly, our work has presented for the first time that both the composition of GW domains and glycosylation modifications of WTA are essential in GW proteins anchoring to the cell wall surface. In addition, we reveal that the crucial roles of autolysin LygA in reinforcing bacteria cross the intestinal barrier and blood-brain barrier, promoting bacterial infection of the host, refining the pathogenic mechanism by which Gal-WTA promotes bacterial infection through retaining the virulence proteins on the *L*. *monocytogenes* surface.

## Methods

### Ethics statement

Animal experiments were performed according to the guide for the welfare and ethics of laboratory animals. All animals were housed in animal biosafety facilities in accordance with the procedures approved by the Institutional Animal Ethics Committee of Yangzhou University (reference number 202106009). All the animals were humanely handled.

### Bacterial strains, cell lines, and animals

The bacterial strains used in this study are listed in S3 Table. LMxysn_1095 encodes a galactosyltransferase (GalT) involved in WTA galactosylation, this gene was deleted to generate Δ*galT* mutant [16]. *L*. *monocytogenes* and *Escherichia coli* strains were cultured in brain heart infusion (BHI; Becton Dickenson, USA) and Luria-Bertani (LB) media at 37°C with continuous shaking at 180 rpm, respectively. The human colon carcinoma enterocyte-like epithelial cell line Caco-2 and human hepatocellular carcinoma cell line HepG-2 were propagated in Dulbecco’s modified Eagle medium (DMEM) supplemented with 10% fetal bovine serum (FBS, Gibco, USA). Six-week-old female BALB/c and C57BL/6 mice were purchased from the Comparative Medicine Center of Yangzhou University (Yangzhou, China) and Vital River Laboratory Animal Technology Co., Ltd (Beijing, China), respectively. All mice were housed in the mouse isolators with SPF conditions (Suzhou monkey animal experiment equipment Technology Co. Ltd., Suzhou, China).

### Construction of mutant and complement strains

The primers used in this study are listed in S4 Table. All the mutant and complemented strains were constructed using the recombinant pAULA plasmid as described previously [16]. The complemented strain Δ*lygA*::*lygA* was obtained on the basis of Δ*lygA*. Replacement of the tag with TTGAAG, instead of TTAAAA, following the termination codon was used to confirm the reversion.

### Polyclonal antibodies production

The plasmids pGEX-6P-I and pET30a were used for the expression of LygA, LygA_GW_, and Auto proteins. The recombinant plasmids were obtained by ligation of the target fragment and vector using the ClonExpress II One Step Cloning Kit (Vazyme, China). Following, the plasmids were introduced into *E*. *coli* strain BL21 (DE3) to generate the protein-expressing strains. BL21 (DE3)-pGEX-6P-I-*lygA*, BL21 (DE3)-pGEX-6P-I-*aut*, and BL21 (DE3)-pET30a-*lygA*_GW_ recombinant strains were induced by IPTG at 37°C for 6 h or at 15°C for 24 h, respectively. The bacterial cells were harvested and disrupted. The supernatant was purified using the GST Fusion Protein Purification Kit to obtain LygA and Auto proteins or the His Bind Purification Kit to obtain LygA_GW_ protein and further analyzed by SDS-PAGE.

Six-week-old female BALB/c mice were immunized with 50 μg purified LygA or LygA_GW_ mixed with an equal volume of Freund’s complete adjuvant for the first immunization and with Freund’s incomplete adjuvant for the subsequent booster immunization after 2 weeks. Blood was collected before the first administration and 14 days after the second administration of protein. Serum was obtained by centrifugation at 1500 ×g for 10 min after clot formation and stored at −20°C.

### LC-MS/MS analysis of surface proteins

The extraction of surface and secreted *L*. *monocytogenes* proteins was performed as described previously [6,19]. The supernatant and bacterial pellets from bacterial cultures (OD_600_ = 0.80) were collected by centrifugation at 9000 rpm for 20 min, respectively. Subsequently, the supernatant was shaken for 1 min after adding a final concentration of 10% TCA and placed overnight at 4°C. Proteins were collected and washed twice with ice-cold acetone. Finally, the secreted proteins were air-dried and dissolved in Tris-HCl (pH 8.0) for analysis by Western blot. On the other hand, bacterial pellet was resuspended with PBS containing 1% SDS and incubated at 37°C for 45 min. After centrifugation, the supernatant was added to a final volume of 10% TCA and placed overnight at 4°C. The following day the samples were centrifuged and dissolved in Tris-HCl (pH 8.0) to analyze by Western blot.

The peptides of surface proteins were analyzed by MS/MS coupled with UPLC through an NSI source. The electrospray voltage applied was 2.0 kV. The scan range was 350–1800 m/z for the full scan, and intact peptides were detected using Orbitrap at a resolution of 70,000. Peptides were then selected for MS/MS using the NCE setting of 28, and the fragments were detected using Orbitrap at a resolution of 17,500. A data-dependent procedure that alternated between one MS scan followed by 20 MS/MS scans with 15.0-s dynamic exclusions was performed. Automatic gain control (AGC) was set at 5E4. The fixed first mass was set at 100 m/z.

The resulting MS/MS data were processed using the MaxQuant search engine (v.1.6.6.0). Tandem mass spectra were searched against the *L*. *monocytogenes_strain_ATCC_BAA-679_169963_PR_20190801* and *L*. *monocytogenes_strain F2365_265669_PR_20191122* UniProt database concatenated with the reverse decoy database. Trypsin/P was specified as a cleavage enzyme, allowing up to four missing cleavages. The mass tolerance for precursor ions was set at 20 ppm in the first search and 5 ppm in the main search. Moreover, the mass tolerance for fragment ions was set at 0.02 Da. The FDR was adjusted to < 1%, and the minimum score for modified peptides was set to > 40. The quantified proteins in each group were analyzed using a two-tailed t-test. *P* < 0.05 and fold-change > 2.0 were defined as significant.

### Western blot analysis

Surface and secreted *L*. *monocytogenes* proteins were analyzed by SDS-PAGE and subsequently transferred into a nitrocellulose membrane for Western blot analysis. Protein concentrations were determined using the BCA Protein Assay Kit (Beyotime, China), and concentrations of 30 μg/well were used for the analysis. Proteins were detected using the mouse polyclonal anti-LygA serum, mouse polyclonal anti-LygA_GW_ serum, mouse monoclonal antibody against ActA (6F5), and mouse monoclonal antibody against LLO (3B6) at 1:2000 dilution. HRP-labeled goat anti-mouse IgG (Calbiochem, Germany) was used as a secondary antibody at 1:8000 dilution. Blots were developed using the ECL Western Blotting Substrate.

### Quantitative RT-PCR

*L*. *monocytogenes* cultures were centrifuged and total RNA was extracted using the RNAprep Pure Cell/Bacteria Kit (Tiangen, China), according to the manufacturer’s protocol. Following this, RNA was reverse transcribed into cDNA using the PrimeScript RT Reagent Kit with gDNA Eraser (Takara, China). qRT-PCR was performed using FSU SYBR Green Master on the QuantStudio 6 Flex System under the following cycling conditions: 50°C for 2 min and 95°C for 10 min, followed by 40 cycles of 95°C for 15 s and 60°C for 1 min. The relative expression level of *lygA* was analyzed by the 2^−ΔΔCt^ method [44] using the subunit B protein of DNA gyrase (*gyrB*) as a reference.

### Bacteriolytic activity analysis

The bacteriolytic activity of LygA was assessed as described previously [45]. *M*. *lysodeikticus* cells (OD_600_ = 0.50) were collected by centrifugation and incubated in 0.1 M EDTA (pH 8.0) for 5 min at room temperature. Then, the sensitized cells were resuspended in the buffer (50 mM Tris-HCl, 10 mM CaCl_2_, pH 7.0) to a final OD_600_ value of 1.5. The cell suspension was incubated with various purified recombinant proteins (10 μg/mL) and an equal volume of PBS (as control) for 30 min, and the decrease in OD_600_ was recorded. The difference in OD_600_ between the experimental and control groups was used to calculate the bacteriolytic activity of the protein. One unit of bacteriolytic activity was defined as the amount of enzyme that decreased OD_600_ at a rate of 0.001/min.

The autolysis assay was performed as described previously [18]. *L*. *monocytogenes* cells in the exponential phase were harvested and washed thrice with precooled distilled water. The cell pellets were resuspended in 50 mM glycine-HCl (pH 8.0) at an OD_600_ value of 1.00. The bacteria were cultured at 37°C, and the OD_600_ value of the bacteria was monitored over time. The autolytic activity was assessed by calculating the ratio of the OD_600_ value to the initial value.

### Growth curve analysis

Bacterial cells were harvested and transferred into small conical flasks containing 10 mL of BHI medium. Three parallel groups were set for each strain and adjusted to an initial OD_600_ value of 0.05. Bacterial cultures were incubated at 37°C, 180 rpm. Then the OD_600_ value of each flask was measured every 2 hours until the bacteria reached to the stationary phase.

### Scanning electron microscopy

The sensitized *M*. *lysodeikticus* cells on glass slides were incubated with different recombinant proteins and fixed with 2.5% glutaraldehyde overnight at 4°C. In addition, exponential-phase bacteria were also grown on glass slides and fixed. All samples were rinsed thrice with 0.1 M PBS; dehydrated in 30%, 50%, 70%, 80%, 90%, 95%, and 100% ethanol solutions for 15 min; and dried in a critical point dryer. The samples were finally gilded using an ion plating apparatus and observed under a GeminiSEM 300 scanning electron microscope.

### Fluorescence microscopy

Overnight bacterial cultures were collected and washed twice with PBS. Bacteria were fixed with 4% paraformaldehyde at room temperature for 20 min and then blocked with PBS containing 5% BSA at 37°C for 2 h. The bacteria were washed thrice with PBS for 5 min each time. Then, the bacteria were successively incubated with LygA or LygA_GW_ polyclonal antiserum and Alexa 488-conjugated goat anti-mouse antibody (Abcam, UK) at 37°C for 2 h. The secondary antibody was replaced with DAPI and incubated for 15 min at room temperature. Finally, the DAPI solution was aspirated and the cells were resuspended in PBS to prepare slides, which were scanned using LAS X software on a Leica-SP8ST-WS computer.

### Surface plasmon resonance (SPR) analysis

The extraction and analysis of WTA were performed as described previously [46]. The binding ability of purified *L*. *monocytogenes* WTA polymers to proteins was determined by SPR analysis as described previously, with minor modifications [17]. The CM5 chip was coated with 0.2 mg/mL LygA, LygA_GW_, or Auto proteins in sodium acetate (10 mM, pH 4.0) at a flow rate of 10 μL/min using amine coupling. Concentrations ranging from 0 to 25 μM of purified WTA as an analyte were flowed through the chip surface in running buffer (10 mM HEPES, 150 mM NaCl, 3 mM EDTA, 0.05% v/v Surfactant P20, pH 7.4) at 10 μL/min at 25°C. Following this, the chip was regenerated by injecting regeneration buffer (glycine-HCl, pH 2.5). For each cycle, the association was measured for 180 s and dissociation was measured for 300 s. For all curves, the 1:1 binding model gave the best fit and was used to calculate the K_D_ values.

### *In vitro* adhesion and invasion assays

Adhesion and invasion assays were performed as described previously [16]. Exponential-phase bacterial cells were pelleted and diluted appropriately in DMEM. Confluent monolayers were infected with bacteria at an MOI of 20 for 1 h. The medium was removed, and the cells were washed thrice with PBS and lysed in 0.2% Triton X-100 for 8 min. The number of bacteria released from the cells was assessed after serial dilutions of the lysates onto agar plates. To assess the invasion rate, the cells were incubated for another 15 min by adding DMEM containing 50 μg/mL gentamicin to kill extracellular bacteria.

### Mouse infection

Six-week-old female C57BL/6 mice (n = 6/group) were used for *in vivo* infection experiments following the intragastric inoculation of *L*. *monocytogenes* XYSN, Δ*lygA*, and Δ*lygA*::*lygA* strains. In brief, the mice were starved for 12 h before treatment (water was allowed) and were challenged with approximately 1 × 10^6^ CFU and 3 × 10^6^ CFU of bacteria at a volume of 0.5 mL (containing 30 mg/mL CaCO_3_) per mouse. Bacterial counts in the spleen, liver, colon, colonic contents and brain were determined on days 1 and 3 post-inoculation. The colon sample was incubated with 20 mL PBS containing 15 mg/mL gentamicin for 30 min to kill any extracellular *L*. *monocytogenes* [47]. Bacterial counts (in CFU) were determined by plating serial dilutions of organ homogenates on BHI agar plates (spleen, liver, and brain samples) or CHROMagar plates (colon and colonic contents samples). Kaplan-Meier plots of six-week-old female C57BL/6 mice (n = 8/group) were used to estimate survival rates following orogastric inoculation with 3 × 10^6^ CFU. The survival of mice was observed and recorded continuously for 14 days.

### Data analysis

Statistical analyses were performed with GraphPad Prism 6 (GraphPad Software, version 6.02). One-way ANOVA with Tukey’s multiple comparisons test was used for pairwise comparison of means from more than two groups. Two-way analysis of variance with Sidak’s multiple comparisons test and Dunnett’s multiple comparisons test were used to compare the means of two or more groups. *P < 0.05, **P < 0.01, ***P < 0.001, ****P < 0.0001 were considered statistically significant. Statistically nonsignificant (ns) was denoted for *P*-values > 0.05.

## Supporting information

S1 Fig Galactosylated WTA promotes surface association of ActA. The surface proteins of *L*. *monocytogenes* obtained from XYSN, Δ*galT*, and Δ*galT*::*galT* strains were analyzed by Western blot using LLO protein levels as a control. Proteins were detected with mouse monoclonal antibody against ActA (6F5) and mouse monoclonal antibody against LLO (3B6). The experiment was repeated twice independently.
(TIF)

S2 Fig Association between WTAs and GW proteins. (A) Alcian blue-stained 20% polyacrylamide gel containing WTA extracted from XYSN, Δ*galT*, and Δ*galT*::*galT* strains. (B, C) SDS-PAGE analysis of purified GST-LygA (99 kDa) and His-LygA_GW_ (54 kDa). (D, E) Assessment of binding kinetics of GST-LygA and His-LygA_GW_ with WTA polymers extracted from XYSN, Δ*galT*, and Δ*galT*::*galT* strains by SPR analysis. RUs: relative units.
(TIF)

S3 Fig GW proteins interact with glycosylation of WTA. (A) SDS-PAGE analysis of purified GST-Auto (86 kDa). (B) The association of Auto with WTA polymers was analyzed through SPR. WTA was extracted from XYSN, Δ*galT*, and Δ*galT*::*galT* strains. RUs: relative units. (C) Assessment of binding kinetics of GST-Auto with WTA polymers extracted from *L*. *monocytogenes* through SPR analysis. RUs: relative units. (D) Analysis of interactions between Auto and WTA at concentrations ranging from 0 to 6.25 μM to determine the binding affinity response. Error bars represent SD. n = 3 independent experiments. Statistical analyses were performed by Tukey’s multiple comparisons test. **P < 0.01; ns: no significance. (E, F) Analysis of binding abilities of LygA and Auto with WTA extracted from EGD-e strain at 3.13 μM.
(TIF)

S4 Fig Comparative analysis of LygA in *L*. *monocytogenes* virulence. (A) and (B) Bacteria load in the organs at 24 and 72 h post-infection. C57BL/6 mice were orogastrically inoculated with XYSN, Δ*lygA* and Δ*lygA*::*lygA* strains at a dose of 1 × 10^6^ CFU. Each dot represents an organ from one infected mouse. Log CFUs/g represents the mean of six mice per group. Error bars represent SD. Data were obtained from two independent experiments. Statistical analyses were performed by Tukey’s multiple comparisons test. *P < 0.05; **P < 0.01; ***P < 0.001; ****P < 0.0001; ns: no significance. (C) The culture of XYSN, Δ*lygA*, and Δ*lygA*::*lygA* was adjusted to the initial OD_600_ = 0.05 in BHI. The bacterial cultures were incubated at 37°C, 180 rpm. The growing curves were measured every 2 hours. Error bars represent SD. n = 3 independent experiments.
(TIF)

S5 Fig Heatmap based on the similarity of GW structure. The GW domains from LygA were aligned and analyzed one by one with those from Auto using BLAST. The numbers represent the degree of similarity between sequences.
(TIF)

S1 Table All the proteins were identified through quantitative proteomic analysis. The UniProt database is *L*. *monocytogenes_strain_ATCC_BAA-679_169963_PR_20190801*. The quantitative information was the relative quantitative value. The LFQ intensity (I) of the protein in different samples was transformed to obtain the relative quantitative value (R) of the protein in different samples (the calculation formula is as follows: Rij = Iij/Mean (Ij), i is the sample, j is the protein), and the number of different proteins in the comparison group at each multiple of difference in the summary was calculated based on the relative quantitative value of the protein.
(XLSX)

S2 Table Proteomics analysis with *L*. *monocytogenes_strain F2365_265669_PR_20191122* UniProt database. (XLSX)

S3 Table Bacterial strains and plasmids used in this study. (PDF)

S4 Table Primers used in this study. (PDF)
