# Supplementary figures and images for: LygA retention on the surface of Listeria monocytogenes via its interaction with wall teichoic acid modulates bacterial homeostasis and virulence
Source: PLoS Pathog. 2023 Jun 28;19(6):e1011482. doi: 10.1371/journal.ppat.1011482 (PMC10335673; doi:10.1371/journal.ppat.1011482)

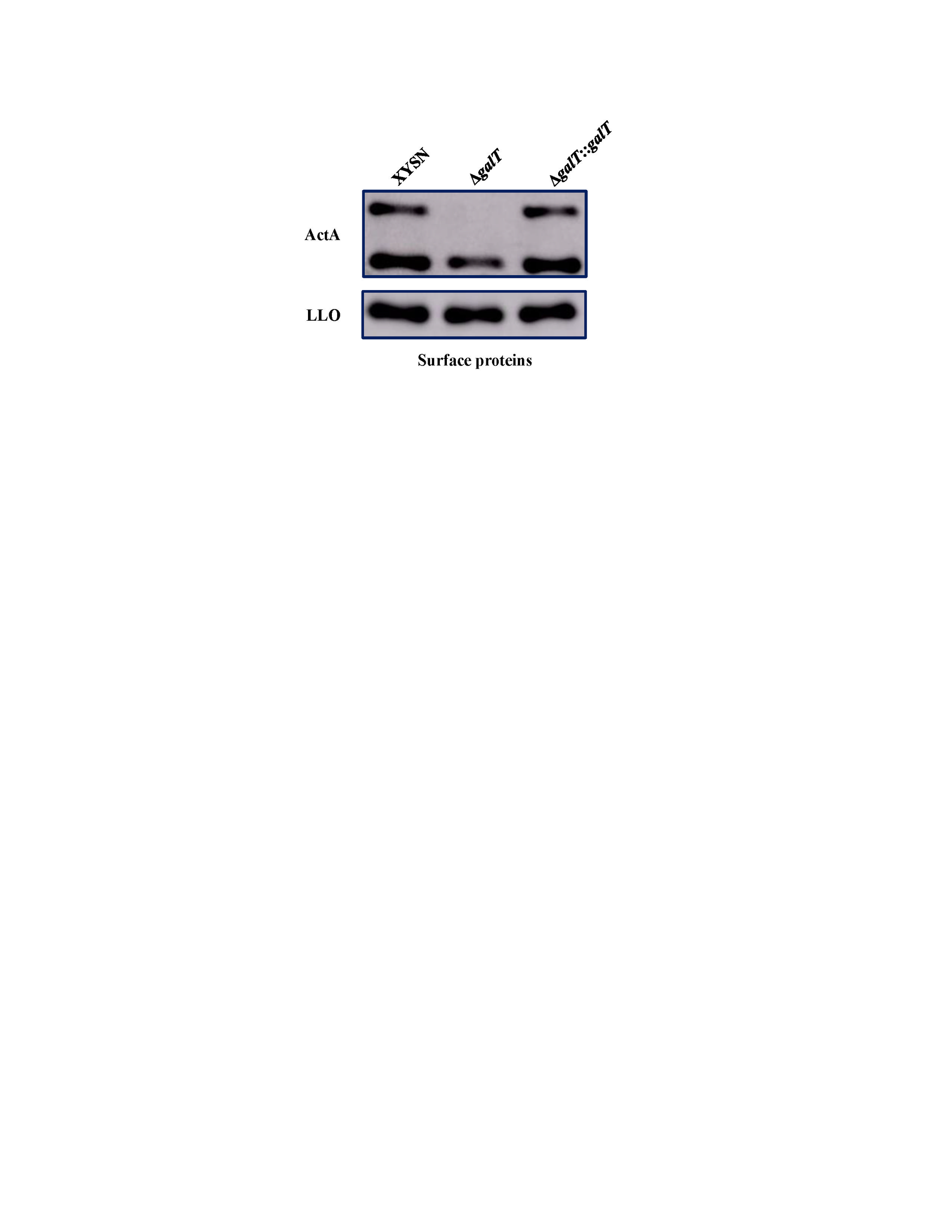

Supplement: S1 Fig — The surface proteins of L. monocytogenes obtained from XYSN, ΔgalT, and ΔgalT::galT strains were analyzed by Western blot using LLO protein levels as a control. Proteins were detected with mouse monoclonal antibody against ActA (6F5) and mouse monoclonal antibody against LLO (3B6). The experiment was repeated twice independently. (TIF) [file ppat.1011482.s001.tif]

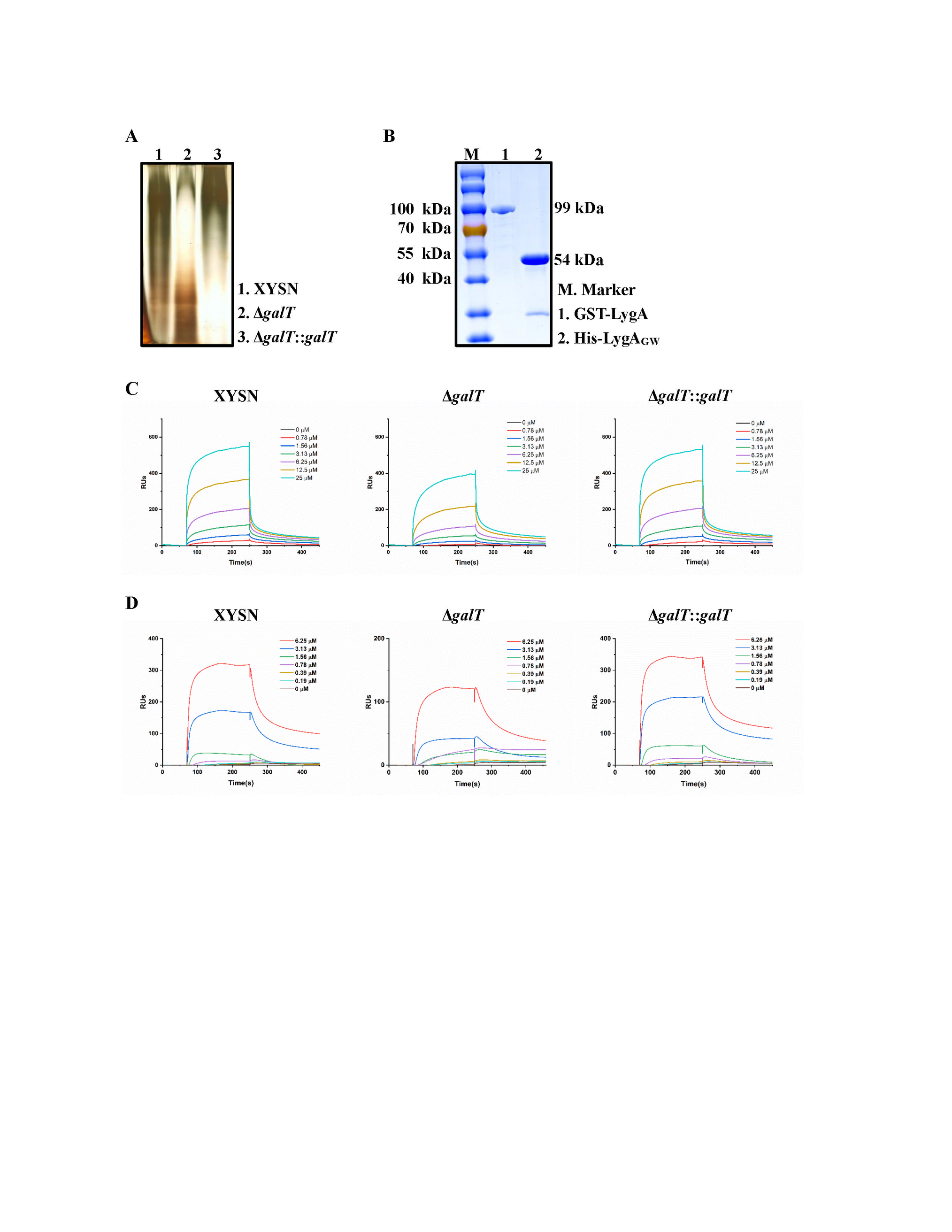

Supplement: S2 Fig — (A) Alcian blue-stained 20% polyacrylamide gel containing WTA extracted from XYSN, ΔgalT, and ΔgalT::galT strains. (B, C) SDS-PAGE analysis of purified GST-LygA (99 kDa) and His-LygAGW (54 kDa). (D, E) Assessment of binding kinetics of GST-LygA and His-LygAGW with WTA polymers extracted from XYSN, ΔgalT, and ΔgalT::galT strains by SPR analysis. RUs: relative units. (TIF) [file ppat.1011482.s002.tif]

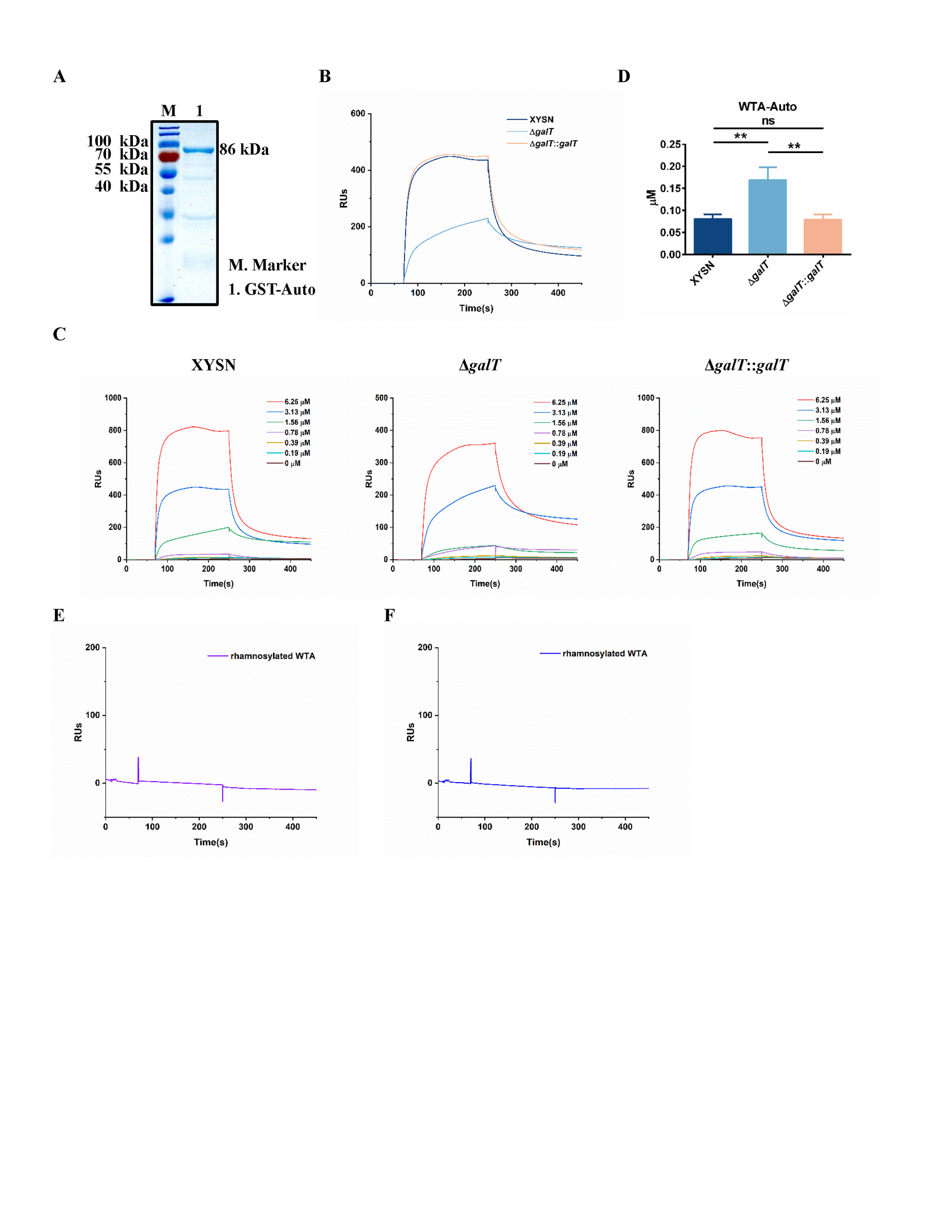

Supplement: S3 Fig — (A) SDS-PAGE analysis of purified GST-Auto (86 kDa). (B) The association of Auto with WTA polymers was analyzed through SPR. WTA was extracted from XYSN, ΔgalT, and ΔgalT::galT strains. RUs: relative units. (C) Assessment of binding kinetics of GST-Auto with WTA polymers extracted from L. monocytogenes through SPR analysis. RUs: relative units. (D) Analysis of interactions between Auto and WTA at concentrations ranging from 0 to 6.25 μM to determine the binding affinity response. Error bars represent SD. n = 3 independent experiments. Statistical analyses were performed by Tukey’s multiple comparisons test. **P < 0.01; ns: no significance. (E, F) Analysis of binding abilities of LygA and Auto with WTA extracted from EGD-e strain at 3.13 μM. (TIF) [file ppat.1011482.s003.tif]

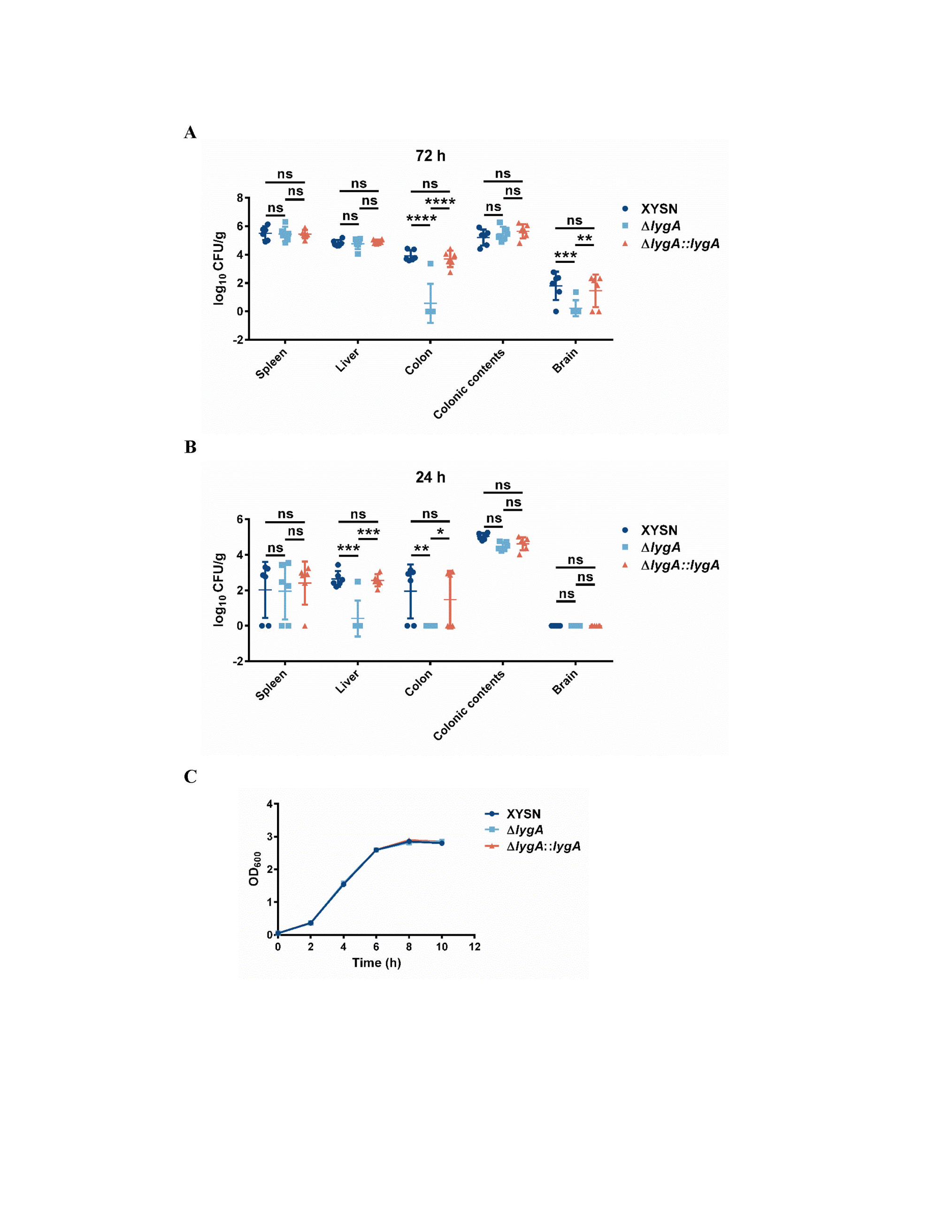

Supplement: S4 Fig — (A) and (B) Bacteria load in the organs at 24 and 72 h post-infection. C57BL/6 mice were orogastrically inoculated with XYSN, ΔlygA and ΔlygA::lygA strains at a dose of 1 × 10^6^ CFU. Each dot represents an organ from one infected mouse. Log CFUs/g represents the mean of six mice per group. Error bars represent SD. Data were obtained from two independent experiments. Statistical analyses were performed by Tukey’s multiple comparisons test. *P < 0.05; **P < 0.01; ***P < 0.001; ****P < 0.0001; ns: no significance. (C) The culture of XYSN, ΔlygA, and ΔlygA::lygA was adjusted to the initial OD600 = 0.05 in BHI. The bacterial cultures were incubated at 37°C, 180 rpm. The growing curves were measured every 2 hours. Error bars represent SD. n = 3 independent experiments. (TIF) [file ppat.1011482.s004.tif]

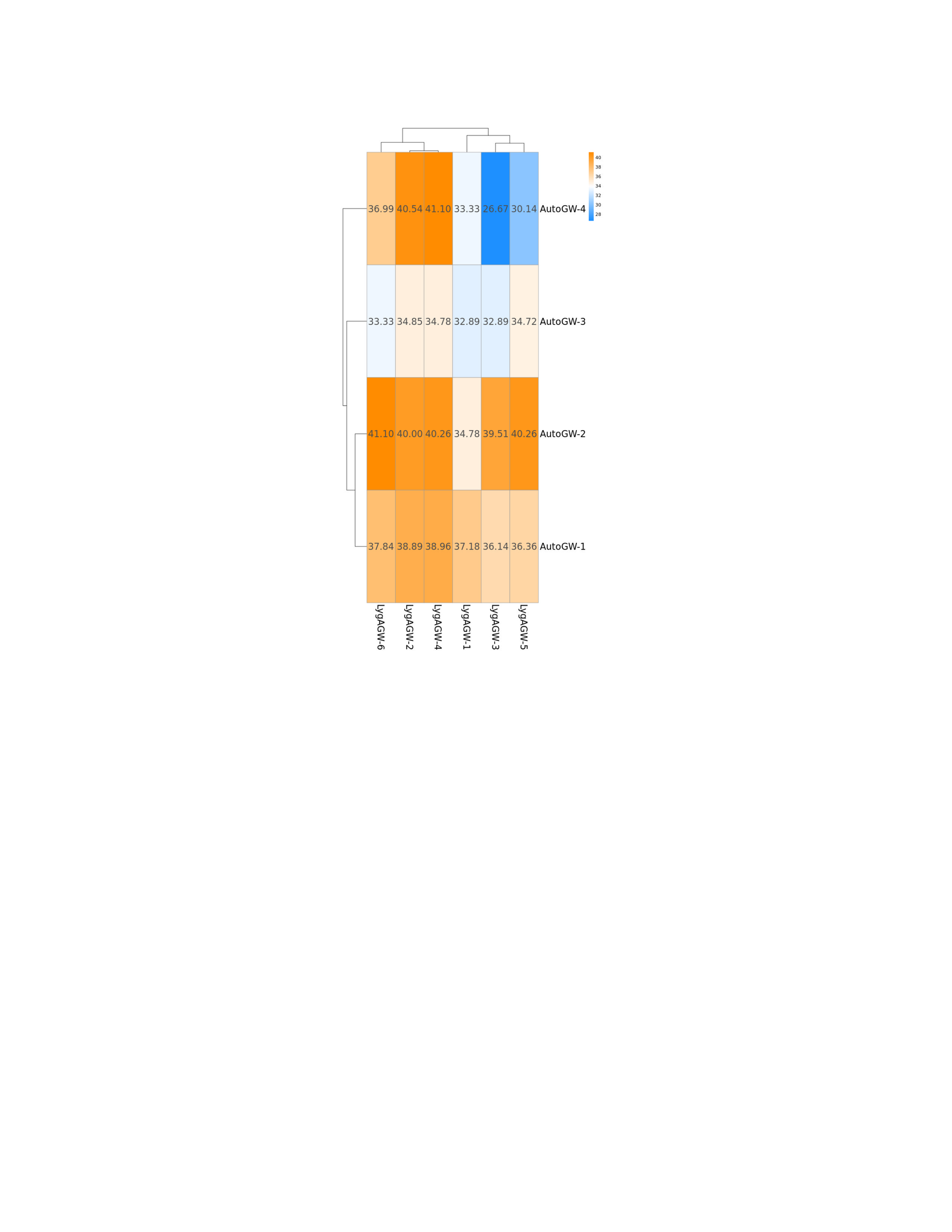

Supplement: S5 Fig — The GW domains from LygA were aligned and analyzed one by one with those from Auto using BLAST. The numbers represent the degree of similarity between sequences. (TIF) [file ppat.1011482.s005.tif]
