# Supplementary material for: LygA retention on the surface of Listeria monocytogenes via its interaction with wall teichoic acid modulates bacterial homeostasis and virulence
Source: PLoS Pathog. 2023 Jun 28;19(6):e1011482. doi: 10.1371/journal.ppat.1011482 (PMC10335673; doi:10.1371/journal.ppat.1011482)
Supplement: S3 Table — (PDF) [file ppat.1011482.s008.pdf]

Supplementary table 3. Bacterial strains and plasmids used in this study

Strains and plasmids	Description	Source
L. monocytogenes XYSN	Wild type, serovar 4h	[1]
L. monocytogenes Δ*galT*	XYSN_1095$_{564\text{-}1791}$ deletion mutant	[1]
L. monocytogenes Δ*galT*::*galT*	Recombinant *galT*$_{564\text{-}1791}$ in XYSNΔ*galT*	[1]
L. monocytogenes EGD-e	Wild type, serovar 1/2a	[1]
Δ*lygA*	*lygA* absents in XYSN	This study
Δ*lygA*::*lygA*	recombinant *lygA* in the Δ*lygA*	This study
Δ*ami*	*ami* absents in XYSN	This study
Δ*lygA*/*ami*	*lygA* and *ami* absent in XYSN	This study
LygA-ΔGW[6]	One GW domain absent of LygA in XYSN	This study
LygA-ΔGW[5-6]	Two GW domains absent of LygA in XYSN	This study
LygA-ΔGW[4-6]	Three GW domains absent of LygA in XYSN	This study
LygA-ΔGW[3-6]	Four GW domains absent of LygA in XYSN	This study
Escherichia coli (DH5α)	Commercial strain used for cloning	Takara Biotechnology (Dalian) Co., Ltd.
Escherichia coli (DE3)	Commercial strain used for expression	Tiangen Biotechnology (Dalian) Co., Ltd.
pGEX-6P-I	Amp^R	This laboratory
pET30a	Km^R	This laboratory
pAULA	Em^R	[1]

References

1. Yin Y, Yao H, Doijad S, Kong S, Shen Y, Cai X, et al. A hybrid sub-lineage of *Listeria monocytogenes* comprising hypervirulent isolates. *Nat Commun*. 2019;10(1):4283. doi: 10.1038/s41467-019-12072-1. PubMed PMID: 31570766; PubMed Central PMCID: PMCPMC6768887.
