# Supplementary material for: LygA retention on the surface of Listeria monocytogenes via its interaction with wall teichoic acid modulates bacterial homeostasis and virulence
Source: PLoS Pathog. 2023 Jun 28;19(6):e1011482. doi: 10.1371/journal.ppat.1011482 (PMC10335673; doi:10.1371/journal.ppat.1011482)
Supplement: S4 Table — (PDF) [file ppat.1011482.s009.pdf]

Supplementary table 4. Primers used in this study

Primers	Sequence (5' to 3')
LygA-F	TTCTGTTCCAGGGGCCCCTGGGATCCATGCAATTACAAGATTCATTAACTGGAGAAA
LygA-R	AGTCAGTCACGATGCGGCCGCTCGAGTCATTTAACATTAGTAAAAGCGCGTTTATC
LygA$_{GW}$-F	TAAGAAGGAGATATACATATGACTCAAAAATCCACTTCTGGAAAAG
LygA$_{GW}$-R	CTCGAGTGCGGCCGCAAGCTTTCATTTAACATTAGTAAAAGCGCGTTTATC
Auto-F	TTCTGTTCCAGGGGCCCCTGGGATCCATGGCTGAAACAACTAATGGAGTAGA
Auto-R	AGTCAGTCACGATGCGGCCGCTCGAGTTAATATTTAAATGCTTTTTTGTCCATC
LygA-P1	AAAACGACGGCCAGTGAATTCCAGGTGTTATTCCTCCGACACAA
LygA-P2	TAATATTCTCCAACCACTCCTTAATTTGT
LygA-P3	GGAGTGGTTGGAGAATATTAAAATAAACCGCCTCCAATTATG
LygA-P4	TGCCTGCAGGTCGACTCTAGATTCCCCTTTCTTCAATCCAGATT
LygA-W1	TATAGGAGGTTAGACAGATGGG
LygA-W2	AAATAATGCGGATACTACTTGG
H-LygA-P1	AAAACGACGGCCAGTGAATTCGGTGATCAGACTTTTTATGAGAAATGTG
H-LygA-P2	GGAGGCGGTTTACTTCAATTATTTAACATTAGTAAAAGCGCGTTT
H-LygA-P3	GCTTTTACTAATGTTAAATAATTGAAGTAAACCGCCTCCAATTATG
H-LygA-P4	TGCCTGCAGGTCGACTCTAGAGACTCGTAATGGTTTAAACAATTTATCTT
Ami-P1	AAAACGACGGCCAGTGAATTCCGGCAGGAGGACACAATGTC
Ami-P2	GCACTAAGGTTCGAAGCGTTTAAATCCTCTCCTAACTGCACTACT
Ami-P3	AACGCTTCGAACCTTAGTGCTACTAAATAA
Ami-P4	TGCCTGCAGGTCGACTCTAGACGTATCGGTTTAGTTGGTAAATATGTT

Ami-W1	CTTTAGCCTCTAAGACGGGAAGA
Ami-W2	GCACCACAAGCGGAAATGACT
GW[6]-P1	AAAACGACGGCCAGTGAATTCGGTTGGATTGATAAACGAGCTCTC
GW[6]-P2	GAGGCGGTTTATTTTAATTATTCAACTGAATCATAAATATCAAAAGCT
GW[6]-P3	TAATTAAAATAAACCGCCTCCAATT
GW[6]-P4	TGCCTGCAGGTCGACTCTAGATCTAATTCCGACTATTTCTTAATCGATT
GW[5-6]-P1	AAAACGACGGCCAGTGAATTCCGGTAAAGTAGTTGGTTGGGTAGAT
GW[5-6]-P2	GAGGCGGTTTATTTTAATTAATTAATATTGTCATATACATCGAAAGCTTT
GW[5-6]-P3	TAATTAAAATAAACCGCCTCCAATT
GW[5-6]-P4	TGCCTGCAGGTCGACTCTAGATCTAATTCCGACTATTTCTTAATCGATT
GW[4-6]-P1	AAAACGACGGCCAGTGAATTCGGTGCTAGCTTAATTCAAGTAATTGAG
GW[4-6]-P2	TTCCTCTTGATCATACAGTGTAATAGCG
GW[4-6]-P3	CACTGTATGATCAAGAGGAATAATTAAAATAAACCGCCTCCAATT
GW[4-6]-P4	TGCCTGCAGGTCGACTCTAGATCTAATTCCGACTATTTCTTAATCGATT
GW[3-6]-P1	AAAACGACGGCCAGTGAATTCGGGAAATATAATGGACAATCAGTTATCA
GW[3-6]-P2	TAAATAATGGAATCATACGGATAGATAGTGA
GW[3-6]-P3	CCGTATGATTCCATTATTTAATTAAAATAAACCGCCTCCAATT
GW[3-6]-P4	TGCCTGCAGGTCGACTCTAGATCTAATTCCGACTATTTCTTAATCGATT
GW-W1	TGTAATGATTCCGATGCTA
GW-W2	TGGGTGGATAATGAAACTG
q-LygA-F	TTGTAATGATTCCGATGC
q-LygA-R	TAAATGCTTGTTGCGATG
gyrB-F	TAATGCTCTTTCCACATCTCTTG
gyrB-R	CGGTAATCCGTTTCGCCT
